# Prognostic influence of witness/victim experiences and PTSD-specific symptoms on working and educational capacity: a comparison between two groups of individuals post-trauma

**DOI:** 10.1186/s12991-015-0045-3

**Published:** 2015-02-12

**Authors:** Helge H Müller, Sebastian Moeller, York Hilger, Wolfgang Sperling

**Affiliations:** Department of Psychiatry and Psychotherapy, Friedrich-Alexander University of Erlangen-Nuremberg, Schwabachanlage 6, D-91054 Erlangen, Germany; Department of Neurology, Friedrich-Alexander University of Erlangen-Nuremberg, Erlangen, Germany; Institution of Statistical Analysis, Freiburg im Breisgau, ᅟ, Germany

**Keywords:** PTSD symptoms, Educational/working capacity, Prognostic outcome

## Abstract

**Background:**

Trauma exposure depends of the type of trauma and can result in the development of posttraumatic stress disorder (PTSD). The type of traumatization (such as Holocaust experiences and other sources of trauma) and specific symptoms of PTSD have influences on the outcome, and specific symptoms of PTSD influence personal and professional outcomes. Another factor is the role of the victim in their traumatization. Some patients are actively traumatized through being victims of torture, while others are passively traumatized by witnessing the traumatization of others.

**Methods:**

We compared two groups of victim/witness trauma sufferers (PTSD vs. Holocaust-experience PTSD (HE-PTSD)) with regard to PTSD symptoms, educational and working capacity, and functional outcome parameters.

**Results:**

HE-PTSD survivors with victim/witness trauma experience showed substantially more specific PTSD symptoms and higher symptom-specific intensities but had high social function and education levels. The intensity and type of intrusive memories and sociodemographic factors do not seem to have a prognostic influence on working or educational outcomes.

**Conclusions:**

Identifying the combined victim/witness experience seems to play an important prognostic role in the assessment of PTSD victims. Further studies should consider these findings within other specific traumatization groups.

## Introduction and background

Exposure to a traumatic event or psychological stress can result in posttraumatic stress disorder (PTSD). PTSD is classified based on five parameters: a person’s exposure to a traumatic event, re-experiencing the event with intrusive memories that are typically reported as “flash-back experiences” by the individual, persistent avoidance of trauma reminders, reduced emotional response after trauma, and persistent symptoms of increased arousal. Education and working abilities and social functioning are impaired in PTSD traumatized persons [1,2].

Moreover, in PTSD, resilience structures are diminished [3]. Resilience is defined as a person’s ability to recover quickly from illness, i.e., the quality that enables something to resume its original shape or position after being bent, stretched, or compressed, and this capacity influences individual psychological elasticity. Resilience is indirectly associated with social functioning and outcome, e.g., educational ability, and thus influences working capacity [4-7].

Patients who have experienced trauma can be divided into groups depending on the type of trauma. One subgroup of PTSD patients is Holocaust survivors (HE-PTSD). Traumatization during the Holocaust was formative for these individuals’ personality and resilience structures [6,8,9]. The main stress factors during the Holocaust experience were early separation from parents, confrontation with the deaths of relatives, physical impairment, and torture [9-11]. Thus, Holocaust survivors suffer from somatic diseases caused by traumatization, and they also experience PTSD-specific symptoms with affective and/or dissociative impairments [1,12,13].

HE-PTSD and PTSD have never been analyzed separately and have not been systematically compared with each other in the context of educational and working capacity outcomes.

## Methods

The comparative study was approved by the local ethical committee and then conducted with 80 HE-PTSD survivors (47 female, 33 male, mean age 76.20 years ± 6.49) and 40 PTSD victims (14 female, 26 male, mean age 46.90 years ± 10.42). All participants gave written consent to participate.

HE-PTSD and PTSD were categorized according to the DSM-IV-R criteria. When evaluating the files, sociodemographic data and sociodemographic outcome parameters, such as gender, age, and school education (years), quality of school education, and years in job were evaluated. Additionally, the type of traumatization (witness or victim); occurrence of flashback-experiences, which were classified by the type of intrusive memory (visual, acoustic, or other); and their intensity (>2/day, >2/week, or >2/month), were analyzed. All participants provided a structured clinical interview to examine (HE-) PTSD-specific symptoms.

For data analysis, we used the statistical program SPSS 22.0, (IBM Corp., Armonk, NY). We tested the data for normal distribution using the Kolmogorov-Smirnov test. For dichotomous variables, the chi-square test or Fisher’s exact test was used, as appropriate. For normally distributed data, variance testing was conducted using Levene’s test. Normally distributed data were compared using *t* tests for unpaired samples. Non-normally distributed data were compared using the Mann-Whitney *U* test, and group differences (e.g., age, gender, and sociodemographic parameters) were tested for significant group bias. For significant group differences, Fisher’s least significant difference (LSD) *post hoc* test was administered. Significance was assumed, consequently, at *p* < 0.05.

## Results

Within the group of Holocaust survivors, all patients (100%) were both victims and witnesses of traumatization. Thirty-four PTSD patients (85%) were only victims (*p* < 0.001), 5 (2%) patients were only witnesses of violent acts against others, and only one patient (0.4%) fulfilled both the victim and witness criteria.

Forty-seven patients (58.8%) of the HE-PTSD group and 31 (77.5%) of the PTSD group had multiple flashbacks or intrusive memory experiences per day. Furthermore, 23 patients (28.8%) in the HE-PTSD group had visual and acoustic intrusive memory experiences, compared to 38 (15.2%) in the PTSD group (*p* < 0.001).

Thirty-seven participants (46.3%) in the HE-PTSD group and 31 participants (77.5%) in the PTSD group had completed basic or primary school; 33 (41.3%) HE-PTSD patients and 5 (12.5%) PTSD-patients had completed high school. Ten (12.5%) HE-PTSD patients and 4 (10%) PTSD-patients obtained university entrance diplomas. There were no significant differences with regard to years of employment between the two groups (*p* = 0.273), (see Table 1).

**Table 1:** **Symptoms, traumatization category, sociodemographic items, and education levels of HE-PTSD and PTSD patients (significance set at <0.05)**

**Parameters**	**HE-PTSD group** **(** ***n*** **= 80)**	**PTSD group** **(** ***n*** **= 40)**	***P*** **value**
Age [years]	76.2 ± 6.5	46.9 ± 10.4	<0.001
Sex [female/male]	47 (58.8%)/33 (41.3%)	14 (35.0%)/26 (65%)	0.020
Traumatization category			
Victim only	0	34	
Witness only	0	5	
Victim and witness	80	1	<0.001
Quality of intrusive memories			
Visual	52	38	
Acoustic	5	0	
Visual and acoustic	23	0	
Other (e.g., olfactory)	0	2	<0.001
Symptom intensity			
>2/day	47	31	
>2/week	26	8	
>2/month	7	1	0.039
School education level			
Basic primary school	37	31	
Secondary modern school			
Junior high school	33	5	
University-entrance diploma	10	4	0.002
Professional qualification			
Labor	40	36	
Mid-level administration	5	1	
Administration	35	3	<0.001
Years in job			
[Mean ± SD]	19.64 ± 19.57	23.83 ± 9.84	0.273

## Discussion and conclusions

Our findings suggest that HE-PTSD survivors have a higher professional qualification level than PTSD patients. One important aspect of PTSD outcome assessment is to consider the symptom complex, which is characterized by the multifactorial influence of traumatization [14-16]. Another important point in the understanding of PTSD symptoms and the associated impairments on work and education capacity is the role of the person within the traumatization. HE-PTSD survivors with high rates of victim and witness trauma experience showed substantially more specific PTSD symptoms and higher rates and different types of symptom-specific intensities. HE-PTSD survivors often show surprisingly high social function and education levels [17], which is also a notable finding in our study. The experience of double traumatization in HE-PTSD, as both witness and victim, is highly significantly associated with higher education levels as a social outcome parameter but not with years of employment compared to PTSD patients.

The other factors examined, such as the intensity and type of reported intrusive memories and sociodemographic factors, such as gender and age, do not seem to have a prognostic influence on work or educational outcomes. It remains unclear whether passive experiences compared to more active victim-witness traumatization are responsible for reduced social functioning and working capacity.

It is a limitation of our examination that both the HE-PTSD and PTSD groups are quite heterogeneous, especially concerning mean age and gender. Thus, the influence of age- and gender-related bias cannot completely be eliminated, although the sample group was controlled by LSD *post hoc* testing, which is the suitable test in random samples of that size. The most remarkable finding is that identification of the combined victim/witness experience seems to play an important prognostic role in the assessment of PTSD victims.

The question arises which other prognostic influence factors, such as medical and psychological treatment, influence the educational and social function levels of PTSD victims and affect personal resilience structures.

Our study is limited by the absence of an extended examination of these potentially important factors in the multifactorial PTSD complex and by differences in the age and gender proportions. It is a natural fact that control groups with equal median ages are difficult to find. There are also more women who survived torture and prosecution within the holocaust experience [18], especially in our sample group. Moreover, we did not assess the time-point of traumatization within the group of PTSD patients.

Further studies with larger participant groups are needed to address these points. Other groups that have experienced other types of trauma should be examined in the context of victim and witness experiences.
